# Targeting Ribosome Biogenesis to Combat Tamoxifen Resistance in ER+ve Breast Cancer

**DOI:** 10.3390/cancers14051251

**Published:** 2022-02-28

**Authors:** Ho Tsoi, Chan-Ping You, Man-Hong Leung, Ellen P. S. Man, Ui-Soon Khoo

**Affiliations:** Department of Pathology, Li Ka Shing Faculty of Medicine, The University of Hong Kong, Hong Kong, China; tsoiho@hku.hk (H.T.); u3006037@connect.hku.hk (C.-P.Y.); george09@connect.hku.hk (M.-H.L.); ellenman@hku.hk (E.P.S.M.)

**Keywords:** breast cancer, c-MYC, ribosome biogenesis, tamoxifen resistance, eIF4E

## Abstract

**Simple Summary:**

Resistance to tamoxifen treatment is an obstacle for ER+ve breast cancer therapy. The overexpression of c-MYC is a known driver of cancer progression and is associated with tamoxifen resistance. Through mediating the up-regulation of ribosome biogenesis and alteration of the transcriptome, c-MYC modulates the translation profile to facilitate the development of tamoxifen resistance. c-MYC is, however, undruggable. Thus, targeting downstream mechanisms mediated by c-MYC might be a more feasible approach. Studies have demonstrated that inhibition of ribosome biogenesis can achieve tumour suppression. Targeting ribosome biogenesis may thus be a feasible strategy to reverse tamoxifen resistance. This article reviews the current evidence to support the feasibility of suppressing ribosome biogenesis to reverse tamoxifen resistance in ER+ve breast cancer.

**Abstract:**

Breast cancer is a heterogeneous disease. Around 70% of breast cancers are estrogen receptor-positive (ER+ve), with tamoxifen being most commonly used as an adjuvant treatment to prevent recurrence and metastasis. However, half of the patients will eventually develop tamoxifen resistance. The overexpression of c-MYC can drive the development of ER+ve breast cancer and confer tamoxifen resistance through multiple pathways. One key mechanism is to enhance ribosome biogenesis, synthesising mature ribosomes. The over-production of ribosomes sustains the demand for proteins necessary to maintain a high cell proliferation rate and combat apoptosis induced by therapeutic agents. c-MYC overexpression can induce the expression of eIF4E that favours the translation of structured mRNA to produce oncogenic factors that promote cell proliferation and confer tamoxifen resistance. Either non-phosphorylated or phosphorylated eIF4E can mediate such an effect. Since ribosomes play an essential role in c-MYC-mediated cancer development, suppressing ribosome biogenesis may help reduce aggressiveness and reverse tamoxifen resistance in breast cancer. CX-5461, CX-3543 and haemanthamine have been shown to repress ribosome biogenesis. Using these chemicals might help reverse tamoxifen resistance in ER+ve breast cancer, provided that c-MYC-mediated ribosome biogenesis is the crucial factor for tamoxifen resistance. To employ these ribosome biogenesis inhibitors to combat tamoxifen resistance in the future, identification of predictive markers will be necessary.

## 1. Introduction

Breast cancer is the most common female malignancy and the second most common cause of cancer death in women. According to the World Health Organization (WHO) (https://www.who.int/news-room/fact-sheets/detail/breast-cancer; assessed on 12 January 2022), 2.3 million women were diagnosed with breast cancer in 2020, with up to 685,000 deaths reported globally. As of the end of 2020, 7.8 million women had been diagnosed with breast cancer in the past five years, making it the world’s most prevalent cancer [1].

The expression of estrogen receptor-α (ER), progesterone receptor (PR), and HER2 are important determinants for treatment and management, with about 70% of breast cancers being ER-positive (ER+ve). ER signalling is the main driving factor for cancer development in ER+ve breast cancer. ER is a ligand-dependent transcription factor. Upon estrogen binding, ER will bind to the responsive element in the target gene promoter, thus regulating the expression of target genes to promote cancer development [2]. This is regarded as the genomic pathway. In addition, ER can participate in the non-genomic pathway, causing the stimulation of the SRC kinase, mitogen-activated protein kinase (MAPK), phosphatidylinositol 3-kinase (PI3K), and protein kinase C pathways in the cytosol [3]. Subsequently, Protein kinase B (AKT) and extracellular signal-regulated kinase (ERK) will be activated, and the activation of these kinases can contribute to tumour development [4]. The established endocrine therapies, namely the selective ER modulators (SERMs) such as tamoxifen, the selective ER down-regulators (SERDs) such as fulvestrant, and aromatase inhibitors (AIs) such as letrozole and anastrozole, have become first-line adjuvant treatments for ER+ve breast cancer. All of them can effectively reduce the rate of breast cancer mortality.

Tamoxifen is the most commonly used adjuvant endocrine therapy. Tamoxifen directly competes with estrogen for binding to ER. In contrast to estrogen, the tamoxifen-receptor complex recruits co-repressors, rather than co-activators, to the promoter regions of estrogen-responsive genes. This blocks their transcription, thus suppressing genomic signalling mediated by ER [5]. Due to the effective antagonism of tamoxifen, it has been shown that tamoxifen can reduce the recurrence rate by around 40% and the mortality rate by around 30% in ER+ve breast cancer patients [6]. However, about 50% of patients who receive tamoxifen will suffer eventual recurrence. Recurrence may be due de novo or acquired resistance to tamoxifen [7]. Thus, resistance presents huge clinical challenges. The dominant mechanism for de novo resistance is lack of ER expression [8], with epigenetic changes in the ER gene contributing in part to this. Alteration in signalling cascades, such as cross-talk between ER and EGFR signalling [9] is an essential mechanism for developing acquired resistance. Alternative splicing (AS), which generates distinct mRNA isoforms from a single gene, also plays an essential role in cancer development and response to treatment [10]. An ER splice variant, ERα36, has been shown to activate ERK1/2 signalling to counter the effect of tamoxifen [11]. A novel splice variant of NCOR2, BQ323636.1, was found to confer tamoxifen resistance by mediating the activation of ligand-independent ER signalling [12]. Although the complete molecular mechanism for endocrine resistance remains to be unraveled, emerging data suggest that c-MYC overexpression may contribute to acquired resistance in ER+ve breast cancers.

## 2. c-MYC in ER+ve Breast Cancer

**Figure 1:**
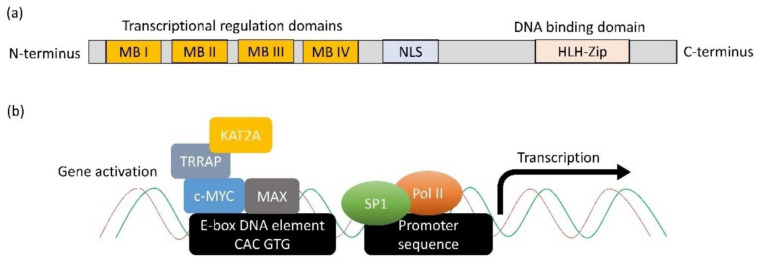
Structure and function of c-MYC. (**a**) The protein structural and functional domains on MYC. (**b**) The molecular mechanism through which c-MYC activates gene expression. c-MYC first dimerises with MAX through the HLH-Zip domain. Next, the dimer binds to DNA via E-box sequence (5′CAC GTG-3′). MB domains on c-MYC recruit other transcription activation factors such as TRRAP and KAT2A to relieve the complex structure of chromatin. Subsequently, the transcription factor, e.g., SP1, can bind to the promoter sequence and recruit DNA polymerase II to initiate transcription.

c-MYC is a transcription factor. It can regulate the expression of genes, controlling the growth and proliferation of cells. It also plays a significant role in enabling tumours to escape immunosurveillance through decreased MHCI expression or upregulation of inhibitory cytokines and immune checkpoint proteins such as PD-L1 and CD47. These provide a compelling rationale for combining c-MYC inhibition with an immune checkpoint blockade [13]. c-MYC inhibition has been demonstrated to cause sustained tumour regression through the promotion of proliferative arrest, differentiation, apoptosis and cellular senescence in cancer cells [14,15]. Together, these data suggest that targeting c-MYC can be exploited as a clinically meaningful therapeutic strategy, making c-MYC one of the most enticing targets for cancer drug development.

c-MYC was discovered almost 40 years ago as the cellular homolog of v-Myc, a viral oncogene derived from an avian myelocytomatosis virus that caused leukaemia and sarcoma in chicken [16]. Two additional human paralogs were eventually identified: MYCN (N-Myc) and MYCL (L-Myc) that can be found in neuroblastoma and lung cancer samples, respectively [17]. The c-MYC protein contains an N-terminal transcriptional regulatory domain, conserved Myc Boxes (MB) I and II, III and IV, followed by a nuclear localisation sequence (Figure 1a). The C-terminus comprises an essential HLH-Zip domain, which interacts with MYC-associated factor X (MAX) to form a dimer that binds DNA and mediates many of its functions [18]. The heterodimer binds to DNA through the binding motif (5′-CAC GTG-3′) termed E-box [19]. Genome-wide localisation analyses and gene expression profiling have revealed that MYC binds to and potentially regulates the transcription of at least 15% of the genome [20,21]. The N-terminal domain has been documented to recruit transcription factors, such as TRRAP, KAT2A, SP1, etc., to form functional transcription complexes to induce the expression of genes (Figure 1b) [22]. Besides activating transcription, c-MYC has been shown to repress transcription by interacting with MIZ1, resulting in the formation of a complex that can recruit DNA methyltransferase 3a (DNMT3a) [23]. In this manner, c-MYC can repress MIZ1 target genes; some of which belong to cell cycle inhibitors such as CDKN1A (p21) and CDKN2B (INK4B) [23,24].

c-MYC is documented to be one of the most up-regulated oncogenes in different types of cancers [25,26]. c-MYC alteration has been reported in 9.92% of breast carcinoma patients [27]. Overexpression of c-MYC has been documented in 30–50% of the poor prognostic cases [28,29,30,31,32]. From the TCGA database (MSK [33]), of 1907 breast cancer cases, there were 1620 that were ER+, 262 that were ER-ve, with 25 of unknown status. Of these 1620 ER+ve breast cancers, amplification of the c-MYC copy number was detected in 7.7% of ER+ve cases (125/1620). Most notable among the c-MYC target genes is the cyclin-dependent kinase CDK4 [34]. Up-regulation of CDK4 is a promoting factor in facilitating cell proliferation. c-MYC is an essential regulator of glutamine and glucose metabolism [35] and appears to affect a broad spectrum of genes that coordinate energy metabolism and biomass accumulation in preparation for DNA replication and cell division in breast cancer.

c-MYC has also been reported to induce the expression of HOXB7, which is a co-factor of ER, to favour the transcriptional activity of ER [36]. c-MYC can suppress the expression of miR-196a, which is an miRNA that represses the expression of HOXB7 [36]. Thus, c-MYC can enhance the activity of ER. Moreover, HOXB7 has been shown to induce the expression of EGFR and its ligands [37]. This suggests that overexpression of c-MYC in breast cancer can activate EGFR signalling. Activation of EGFR signalling can favour tumour progression and the development of tamoxifen resistance [38]. Clinical studies suggest that ER+ve breast cancer patients with overexpression of EGFR have poor survival outcomes and are less likely to benefit from tamoxifen [39]. These findings support the hypothesis that the overexpression of c-MYC will favour the development of breast cancer.

c-MYC can regulate epithelial-to-mesenchymal transition (EMT) necessary for cellular invasion and migration, and thus metastasis. Studies report that c-MYC promotes TGFβ-mediated activation of the SNAIL transcription factor, both directly and indirectly, through a microRNA network involving a LIN28B/let-7/HMGA2 cascade to facilitate metastasis [40,41]. c-MYC can also regulate cell-cell and cell–matrix interactions through transcriptional activation of LGALS1, which is a β-galactosidase binding protein that promotes cell migration and invasion [42,43]. It has been reported that c-MYC also functions with SKP2 to recruit MIZ1 and p300 into a transcriptional complex which activates RhoA, which is necessary for migration, invasion, and metastasis [44]. These studies highlight the importance of c-MYC in breast cancer metastasis.

c-MYC is an estrogen-responsive gene [5]. The overexpression of c-MYC is implicated in resistance to endocrine therapy in ER+ve breast cancer [45,46,47]. Clinical studies have demonstrated that the overexpression of c-MYC could be predictive of shorter time-to-recurrence following the adjuvant tamoxifen [47]. c-MYC can regulate the expression of survivin (BIRC5), which is a member of the inhibitor of apoptosis protein (IAP) family, playing an essential role in tumorigenesis [48,49]. Tamoxifen eradicates ER+ve breast cancer cells by inducing apoptosis to prevent local recurrence and distance metastasis [50]. Overexpression of c-MYC leads to the up-regulation of anti-apoptotic proteins in cancer cells. Therefore, it is not surprising that overexpression of c-MYC can compromise the effect of tamoxifen.

## 3. The Role of c-MYC on Ribosome Biogenesis

**Figure 2:**
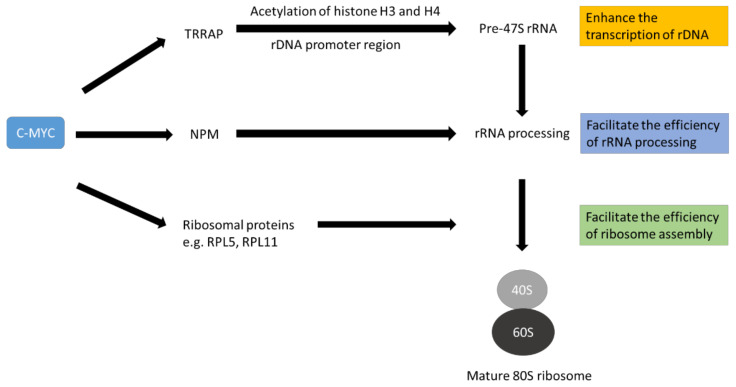
Schematic diagram to illustrate the role of c-MYC in promoting ribosome biogenesis. C-MYC favours the expression of ribosomal proteins, other factors necessary for rRNA processing and ribosomal RNA (rRNA). The ribosomal proteins and other factors help process immature rRNA to mature rRNA. Mature rRNA complexes with ribosomal proteins to form 40S ribosome and 60S ribosome subunits. Finally, an 80S mature ribosome is generated. This complex process is called ribosome biogenesis. The diagram is simplified for illustrating the concept only.

c-MYC can regulate various cellular metabolisms, including glucose and glutamine, which are essential for generating enough energy and intermediates of macromolecules to support the high rate of cancer cell proliferation [51]. In addition, c-MYC has been shown to be involved in ribosome biogenesis, which is a process to produce ribosomes [52,53]. Ribosomes are responsible for translating information contained in mRNAs into functional proteins. It is the ultimate step in the genetic program of translation [54]. The eukaryotic 80S ribosome consists of a small 40S subunit and a large 60S subunit. The 40S subunit is comprised of the 18S ribosomal RNA (rRNA) and 33 different ribosomal proteins, whereas the 60S subunit consists of 25S, 5.8S, and 5S rRNA and 47 ribosomal-proteins [54]. The hyper-activation of ribosome biogenesis initiated by oncogenes or the loss of tumour suppressor genes has a critical effect on cancer initiation and progression [55]. To control ribosome biogenesis, mammalian cells have developed tumour suppressor-based surveillance mechanisms, such as TP53, that can regulate cell proliferation in the event of uncontrolled ribosome production [56]. TP53 can inhibit the RNA pol I transcription machinery to block rRNA synthesis in order to maintain genomic and cellular homeostasis [57]. c-MYC has been shown to repress TP53 by c-Myc-Inducible Long non-coding RNA Inactivating P53 (MILIP) that promotes p53 poly-ubiquitination and degradation by reducing p53 SUMOylation through suppressing tripartite-motif family-like 2 (TRIML2) [58]. In this manner, c-MYC can counteract the tumour suppressive role of TP53 by reducing its availability. Thus, c-MYC removes the brake for rRNA transcription to enhance ribosome biogenesis. Enhanced ribosome biogenesis is necessary to sustain increased protein synthesis and a high proliferation rate in cancer cells. The overexpression of c-MYC can meet these requirements. Recent studies have demonstrated that drugs that inhibit ribosome biogenesis or that target c-MYC might offer a viable therapeutic approach for cancer treatment [59].

c-MYC has been reported to enhance ribosome biogenesis by various mechanisms (Table 1). First, c-MYC increases the transcription of many ribosomal proteins, translation initiation factors and elongation factors through RNA polymerase II [60,61,62]. Suppression of c-MYC expression in a mouse model of osteosarcoma has been shown to reduce the expression of many ribosomal protein genes [63]. In addition, c-MYC can coordinate the transcription of genes that encode proteins required to process rRNA precursors that contribute to ribosome assembly and the nucleocytoplasmic transport of mature ribosomal subunits [60,64,65]. c-MYC can enhance the expression of nucleophosmin, (NPM1) which is a crucial factor in ribosome biogenesis [66]. NPM1 is involved in multiple steps of ribosome biogenesis, including rRNA processing, ribosomal protein stability and the transport of ribosomal subunits into the cytoplasm [67,68]. Furthermore, c-MYC can up-regulate the expression of TRRAP, which is part of a histone acetyltransferase complex. The complex can increase the acetylation of histones H3 and H4 in the ribosomal DNA (rDNA; the gene encode for rRNA) promoter region to favour the transcription of pre-ribosomal RNA [69,70]. Therefore, overexpression of c-MYC can enhance the production of raw materials and essential factors necessary for ribosome biogenesis (Figure 2). The enhanced ribosome biogenesis sustains the high demand of cancer cells for proteins and other building blocks.

On the other hand, the depletion of c-MYC has been shown to diminish ribosome production in colon cancer [71]. Knockdown of c-MYC could reduce the expression of ribosomal protein L5 (RPL5) and L11 (RPL11) [71]. Studies have demonstrated that ribosomal proteins are stabilised by their interaction with rRNAs [72,73]. Depletion of c-MYC can reduce the transcription of rRNA. The reduced availability of rRNA would lead to the reduction of ribosomal proteins due to protein degradation mediated by the proteasome in the nucleus [74]. The ubiquitin ligase Tom1 collaborates with the E2 enzymes Ubc4 and Ubc5 to mediate the degradation of unassembled ribosomal proteins [75], reducing the raw building blocks of the ribosome and thus the mature ribosomes.

## 4. The Effect of c-MYC on Tamoxifen Resistance

c-MYC can induce tamoxifen resistance through various mechanisms. For example, c-MYC has been reported to induce the transcription of survivin (encoded by the gene BIRC5), an essential member of the inhibitor of apoptosis protein (IAP) family, which plays an essential role in tumorigenesis [48]. Through expression analysis, c-MYC has been reported to regulate the expression of nearly 15% of global genes [76], including some commonly regulated by ER. Therefore, the role of ER can be partially replaced by c-MYC [77]. Genes repressed by tamoxifen would thus be induced by c-MYC overexpression. This would abolish the effect of tamoxifen because the genes would no longer be regulated by ER but by c-MYC. Therefore, c-MYC can induce tamoxifen resistance through transcriptional regulation.

In addition, c-MYC can induce tamoxifen resistance via the mRNA translation mechanism. c-MYC has been known to regulate mRNA translation through inducing the expression of translation initiation factors, such as eIF4E, eIF2α, eIF4AI and eIF4GI, which are needed for CAP-dependent translation [78]. c-MYC can directly promote methylation of the mRNA CAP structure through RNA guanine-7-methyltransferase (RNMT), which is essential for CAP binding to eIF4E and the recruitment of the 40S ribosome subunit, thereby favouring cap-dependent mRNA translation [79]. c-MYC can promote eIF4F-dependent translation and cooperate with elF4E to drive tumorigenesis in vivo [80]. Translation of mRNA begins with recognising the 7-methylguanylate-capped structure by the translation initiation complex composed of the cap-binding protein eIF4E, eIF4A, and eIF4G to start translation [81]. Overexpression of eIF4E can increase the efficiency of translating mRNAs containing structured 5′-untranslated region (5′-UTR) [82]. Around 10% of cellular mRNAs have atypically long 5′-UTR. Many encode proto-oncogenes, anti-apoptotic proteins and growth factors [83,84]. A long 5′-UTR and GC-rich sequence form a stable secondary structure. It has been demonstrated that the translation of mRNA with long 5′-UTR is often sensitive to the expression level of eIF4E [85].

The activity of eIF4E is governed by phosphorylation, especially Ser209 [86]. MNK1 and mTORC1 have been demonstrated to modulate the phosphorylation on this site [87]. The study showed that overexpression of eIF4E and its phosphorylation mediated by MNK1 could rewire the translation profile and thus the protein expression profile. RUNX2 was identified as the critical factor, being up-regulated by the overexpression of phosphorylated eIF4E. RUNX2 as a member of RUNX transcription factors involved in lineage-specific cell fate determination [88], regulates gene expression by functioning as a molecular scaffold to recruit chromatin remodelling enzymes (e.g., SWI/SNF and CTCF), and thus modulates promoter accessibility [89]. RUNX2 has been shown to regulate the expression of genes involved in WNT/β-catenin and TGF-β signalling [90]. These two key pathways have been reported to be dysregulated in many cancers, such as breast cancer. In vitro study has shown that suppressing WNT signalling by a small molecule ICG-001 or β-catenin siRNA could reverse tamoxifen resistance [91]. It has been established that TGF-β can activate various signalling pathways, including ERK1/2 and p38MAPKs and PI3K signallings, forming transduction signalling networks [92]. Activation of these signallings is known to contribute to the development of tamoxifen resistance [93]. Therefore, the overexpression of c-MYC, which can enhance these signallings, can thus contribute to the development of tamoxifen resistance in breast cancer.

**Figure 3:**
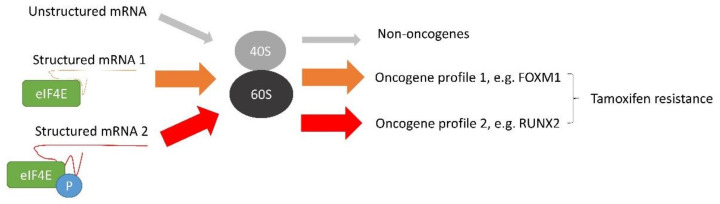
The molecular mechanism mediated by eIF4E to induce tamoxifen resistance in breast cancer. Overexpression of c-MYC enhances the expression of eIF4E that prefers to interact with structured mRNA and favours its translation. mRNAs of oncogenic factors are usually structured. Therefore, eIF4E will favour the protein expression of oncogenic factors. In addition, the activity of eIF4E is regulated by phosphorylation. Phosphorylated and unphosphorylated eIF4E (p-eIF4E) have different selectivity toward structured mRNA. eIF4E and p-eIF4E induce differential protein expression profiles. Therefore, eIF4E and p-eIF4E modulate tamoxifen resistance through different mechanisms.

The overexpression of non-phosphorylated eIF4E can also confer tamoxifen resistance [83]. By investigating translation and polysome fractionation profiles, FOXM1 was found to be significantly up-regulated at the protein level but not at the mRNA level [83]. FOXM1 is an oncogenic transcription factor [94]. Studies have demonstrated that down-regulation of FOXM1 favours apoptosis [95,96]. Thus, upregulation of FOXM1 can counteract the effect of tamoxifen’s effect on apoptosis in breast cancer. Similarly, overexpression of eIF4E can enhance the translation of c-MYC and cyclin D1 [83]. It suggests that c-MYC and eIF4E have a positive feedback mechanism to support the development of tamoxifen resistance. Thus, eIF4E can employ different ways to modulate tamoxifen resistance (Figure 3), and targeting individual signalling might be ineffective to combat the resistance in breast cancer cells.

## 5. Feasibility of Targeting Ribosome Biogenesis Enhanced by c-MYC Overexpression to Reverse Tamoxifen Resistance in Breast Cancers

Aberrant increases in nucleolar size and number that reflects increased ribosome biogenesis has been recognised for over a century as the hallmark of many cancers and has been associated with poor prognosis [97]. Hyper-activation of ribosome biogenesis by c-MYC overexpression can increase global protein synthesis rates, decrease translational fidelity or alter the pattern of translated mRNAs [98]. These contribute to tumorigenesis. A study demonstrated that the down-regulation of ribosome protein RPL24 could reduce the rate of ribosome biogenesis and improve disease-free survival in the mouse cancer model with MYC overexpression [98]. The down-regulation of ribosomal proteins RPL6 [99] and RPS21 [100] has also been reported to suppress cancer cell proliferation. Overexpression of RPL34 was found to increase apoptosis resistance of cancer cells [101]. These studies highlight the importance of ribosomal protein in cancer development. One recent study demonstrated that genes coding for ribosomal proteins and regulators of translation could promote metastasis of breast cancer [102]. An independent bioinformatics study revealed that amplification of RPL8, RPL19, and RPL23 genes was associated with poor survival outcomes in breast cancer [103]. Overexpression of these ribosome proteins might enhance ribosome biogenesis, supporting the development of breast cancer. The elevated expression of RPL23 was shown to confer resistance to apoptosis [104]. Thus, suppressing ribosome biogenesis might reduce aggressiveness and enhance the response of breast cancer to tamoxifen.

c-MYC is a crucial regulator of protein biosynthesis. Target genes of c-MYC mediating transcription are essential for crucial steps in the ribosome biogenesis process, including the synthesis of ribosomal RNAs and proteins [25,105]. The up-regulation of these components is one of the most consistent gene expression signatures associated with c-MYC overexpression [35]. Compounds such as KJ-Pyr-944 or SaJM589 have been shown to suppress cell proliferation by disrupting c-MYC-MAX heterodimerisation and potentially promoting proteasome-mediated c-MYC degradation in leukemic cell lines [106]. Another recently identified small compound, MYCMI-6, also inhibits c-MYC-MAX heterodimerisation by binding to the bHLH-LZ domain of c-MYC and abolishing c-MYC-mediated transcription in breast cancer cells [107]. An in vitro study confirmed that MYCMI-6 could suppress c-MYC-dependent cell growth, which correlates with the level of c-MYC expression in tumour cells. The chemical KSI-3716, which blocks c-MYC-MAX binding to DNA, can also suppress cancer cell proliferation in leukaemia [108]. However, there is no active clinical trial evaluating c-MYC inhibition mediated by the above chemicals on breast tumour suppression. Severe side effects in animal studies might be a problem. Omomyc inhibits c-MYC in part by promoting its proteasomal degradation in lymphoma cell lines [109]. Omomyc is a 90 amino acid c-MYC mutant that comprises the bHLH-LZ domain and competes with c-MYC to bind DNA. It displaces the c-MYC/MAX heterodimers and inhibits the transcription of target genes. Omomyc has been shown to have a potent anti-proliferative effect with sustained tumour regression with no detrimental effect on healthy tissue, thus for the first time, c-MYC inhibition can be considered as a feasible therapeutic anti-cancer strategy [110]. A clinical trial (Phase I/II; NCT04808362) started since 2021 has made Omomyc the first c-MYC inhibitor to reach clinical study in patients with advanced solid tumors, including breast cancer.

A research group demonstrated that the novel small molecule inhibitor CX-5461 could specifically target rDNA transcription, leading to the suppression of tumour development. This chemical has been used in phase 1 clinical trials (NCT02719977; Australia and New Zealand Clinical Trials Registry, #12613001061729) [111,112]. In addition, CX-3543, another small molecule inhibitor to target rDNA transcription, has been shown to suppress cancer successfully and has been used in clinical trials (NCT00955786; NCT00780663; NCT00780663) [113,114]. Haemanthamine, a natural alkaloid extracted from Daffodil bulbs, has been shown to target ribosome function and ribosome production by inhibiting RNA processing specifically to suppress cancer cell proliferation and induce apoptosis in colon cancer and leukemic cancer cells with c-MYC overexpression [115,116]. A pre-clinical model of c-MYC-driven lymphoma xenograft demonstrated that combining CX-5461 with an mTOR inhibitor could effectively suppress tumour growth [117]. Moreover, combining CX-5461 and the pan-PIM-kinase inhibitor CX-6258 has been shown to have an effective tumour-suppressive effect in the abiraterone-and enzalutamide-resistant prostate cancer PDTX model [118]. These studies illustrate the feasibility of targeting ribosome biogenesis in cancer therapy.

Tamoxifen is commonly used for treating ER+ve breast cancer. However, tamoxifen resistance hinders the usefulness of tamoxifen. Identification of agents to reduce the resistance is urgently needed. The overexpression of c-MYC has long been suggested to be one of the significant factors for triggering the resistance [49,119,120]. However, targeting c-MYC with clinical-grade small molecules is still challenging, particularly at the protein level [15]. The protein domains of c-MYC are intrinsically disordered and lack an enzymatically active site. This has posed difficulties in drug design [15]. Due to these difficulties, there is no small molecule c-MYC inhibitor that can be used clinically in the market. Hence, directly inhibiting c-MYC to reverse tamoxifen resistance in breast cancer is not feasible.

The overexpression of c-MYC can enhance ribosome biogenesis to sustain the demand from cancer cells. Therefore, targeting the steps in ribosome biogenesis instead of c-MYC may provide new cues for reducing tamoxifen resistance in breast cancer. CX-5461 [112], CX-3543 [114] and haemanthamine [115], have been shown to repress the efficiency of ribosome biogenesis. CX-5461 has been shown to work with the mTOR inhibitor or the pan-PIM inhibitor to synergise cancer therapy. However, none of these chemicals has been tested for recovering tamoxifen sensitivity in ER+ve breast cancer. A current ovarian cancer study demonstrates that CX-5461 can enhance the efficacy of the PARP inhibitor [121], suggesting the feasibility of using ribosome biogenesis suppression in combination with other drugs to improve drug effectiveness. Different mechanisms can trigger tamoxifen resistance in ER+ve breast cancer, such as mutations [122] and alteration of alternative splicing [12], etc. It would be necessary to determine to what extent c-MYC overexpression and the resultant enhanced ribosome biogenesis contribute to tamoxifen resistance in ER+ve breast cancer. A predictive marker to determine the degree of involvement of ribosome biogenesis in resistant cases can help identify which cases will be responsive to ribosome biogenesis suppression to reverse tamoxifen resistance.

## 6. Conclusions

Breast cancer is a heterogeneous disease. Different subtypes will result in a different response to a particular treatment. Having a single therapy that can be used universally is unrealistic. Similarly, various molecular mechanisms, such as mutations and altered gene expression, can contribute to tamoxifen resistance; some even cross-talk with each other to make the tamoxifen-resistant mechanism more complicated. Therefore, it is not feasible to rely on a single agent to reverse tamoxifen resistance in all patients. c-MYC overexpression is a driver of cancer and drug resistance. The overexpression of c-MYC can rewire the gene expression profile to sustain the demands of cancer cells, enhanced ribosome biogenesis being one of them. Since c-MYC is undruggable, the downstream mechanisms mediated by c-MYC become the targets. Ribosomes are the place to produce proteins for supporting cell proliferation and combating cell death pathways induced by therapeutic agents. The suppression of ribosome biogenesis will therefore compromise the survival advantages of cancer cells. On the one hand, it will suppress the development of cancer cells; on the other hand, it will make the cancer cells more sensitive to the drug or even reverse drug resistance. Therefore, in ER+ve breast cancer, we believe that targeting ribosome biogenesis will be a feasible strategy to deal with tamoxifen resistance.

## Figures and Tables

**Table 1:** Factors that are up-regulated by c-MYC to favour ribosome biogenesis.

Functional Roles	Candidate Proteins	References
Structural proteins of ribosomes	RPL3, RPL6, RPL23, RPL35, RPL44, RPS3	[61]
	RPS19, RPS17, RPS11, RPS24	[60]
	RPL24, RPS11, RPS21, RPS25, RPL10a, RPS24, RPL6, RPL36a, RPS27, RPL3, RPS5	[63]
	RPL5, RPL11	[71]
Factors to facilitate rRNA processing	Fibrillarin (FBL)	[60]
	Nucleolin (NCL)	[61]
	Nucleophosmin (NPM1)	[66]
rDNA transcription	TRRAP	[69]
